# Functional electrical stimulation and brain-machine interfaces for simultaneous control of wrist and finger flexion

**DOI:** 10.1186/s12984-026-02091-7

**Published:** 2026-08-05

**Authors:** Matthew J. Mender, Ayobami L. Ward, Luis H. Cubillos, Madison M. Kelberman, Joseph T. Costello, Hisham Temmar, Dylan M. Wallace, Edanjen T. Lin, Jordan L. W. Lam, Matthew S. Willsey, Nishant Ganesh Kumar, Theodore A. Kung, Parag G. Patil, Cynthia A. Chestek

**Affiliations:** 1 Department of Biomedical Engineering, University of Michigan, Ann Arbor, MI USA; 2 Department of Neurosurgery, University of Michigan Medical School, Ann Arbor, MI USA; 3 Robotics Institute, University of Michigan, Ann Arbor, MI USA; 4 Department of Electrical Engineering, University of Michigan, Ann Arbor, MI USA; 5 Department of Mechanical Engineering, University of Michigan, Ann Arbor, MI USA; 6 Section of Plastic Surgery, Department of Surgery, University of Michigan Medical School, Ann Arbor, MI USA

**Keywords:** Brain-machine interface, Functional electrical stimulation, Spinal cord injuries

## Abstract

**Background:**

Restoring dexterous hand movements in individuals with cervical spinal cord injury requires continuous control of multiple biomechanically linked degrees-of-freedom (DOF). Brain-machine interface (BMI) controlled functional electrical stimulation (FES) is a promising method to restore hand movements. We explored the suitability of restoring movement to two biomechanically linked DOF simultaneously, finger flexion and wrist flexion, using previously introduced FES methods, namely stimulation targeting individual DOFs independently. We then demonstrate a finger and wrist movement BMI that, when combined with the FES system, could restore simultaneous control of wrist and finger flexion.

**Methods:**

Two monkeys were implanted with intramuscular electrodes in muscles of the hand. Stimulation on these electrodes was used to evoke finger and wrist movements in both monkeys. Additionally, one of these monkeys was implanted with intracortical electrode arrays in the primary motor cortex and used a BMI to control wrist and finger flexion in a virtual hand without FES.

**Results:**

First we demonstrate a monkey using an intracortical BMI to control the wrist and finger flexion in a virtual hand, both before and after the hand is temporarily paralyzed, achieving success rates and acquisition times equivalent to able-bodied control with BMI control after temporary paralysis in two sessions. We then show that intramuscular FES with two predetermined stimulation patterns, one targeting finger flexion and one targeting wrist flexion, can move the monkeys’ fingers and wrist across a functional range of motion. Due to the biomechanical coupling of the wrist and fingers, stimulation targeting finger or wrist flexion individually ultimately evokes movements in both DOF. However, we demonstrate that a simple control strategy treating each DOF independently was able to control both DOF simultaneously in a closed-loop task, achieving greater than 80% success rates.

**Conclusions:**

We outline a method using an artificial brain-to-body interface that could restore continuous wrist and finger movements after spinal cord injury.

**Supplementary Information:**

The online version contains supplementary material available at https://doi.org/10.1186/s12984-026-02091-7.

## Introduction

Spinal cord injuries (SCI) impact a person’s ability to perform activities of daily living and negatively impact quality of life, especially when hand function is affected. Almost 60% of SCI cases in the United States are partial or complete injuries at the cervical level which typically decreases hand and arm function [1]. Restoring hand function is a top priority for people with SCI to improve quality of life [2, 3]. Functional electrical stimulation (FES) can restore hand movements after SCI by electrically activating a person’s own muscles. Implanted FES has been used to restore hand movements since at least the 1980s [4] and prescribed movements such as lateral and palmar grasps have been restored in more than 220 users [5]. While restoring prescribed grasps effectively improved patients’ abilities to perform functional tasks, activities of daily living involving the hand often require more dexterous movements involving more degrees-of-freedom (DOF) [6]. To improve the dexterity of restored movement, we studied the simultaneous restoration of 2-DOFs, wrist flexion, and grasping. Restoring control of wrist flexion with grasping would enable users to stabilize their hand, reach new objects, augment the grasp itself [7, 8], and move grasped objects.

Restoring hand control can be broken down into two parts: first, estimating the intended movements of the hand, and second, stimulating to restore multiple-DOF movements. Implemented FES control schemes have determined movement intentions using residual movements [5, 9, 10] and muscle activations [11–14]. While successful, these methods may be unintuitive when scaling to more DOF and may not function in more severe injuries. Brain-machine interfaces (BMIs) provide an intuitive control signal using an intact part of the natural motor control pathway [15]. With improving technologies and algorithms, BMIs have reached unprecedented levels of success in estimating user intentions for various applications [16–20]. Critically, intracortical BMIs have been used to decode intended movements of multiple-DOF in the hand, for example decoding multiple-DOF of the upper limb at the same time during prehensile tasks [20, 21], and multiple continuous DOF movements in the hand [22–25]. As such, intracortical BMIs are promising for decoding continuous finger and wrist flexion continuously.

Intracortical BMIs have been adapted for use with FES showing restoration of upper limb function after paralysis in monkeys [26–30], and in people [25, 31, 32]. The current state of the art brain-controlled FES systems have decoded neural activity into continuous joint angles and then use predetermined mappings (stimulation patterns) to translate intended movements in individual DOFs, such as elbow flexion, wrist flexion, or grasping, into the stimulation parameters required to move that DOF. This method is thought to be more intuitive than controlling individual muscle activations [28] and can extend to more movements than decoding discrete postures [32]. Currently, restored control has been 1-DOF in the hand at a time and has not yet scaled to biomechanically coupled movements such as the wrist and fingers.

Fully restoring movements to both the wrist and fingers at the same time is expected to be difficult because these joints are biomechanically linked. Specifically, the muscles that control the fingers also cross the wrist joint, both changing length with wrist posture and evoking moments of the wrist when moving fingers. With respect to controlling wrist posture in FES systems, studies have stabilized the wrist with splints [33, 34], or tendon transfer from brachioradialis to Extensor Carpi Radialis Brevis [35]. Subsequent iterations of the freehand system augmented the stimulation patterns by including wrist muscle stimulation to stabilize the wrist during grasping [35]. Alternatively, Ajiboye et al. [25], defined a separate stimulation pattern for the wrist flexion DOF which could act independently of the finger flexion DOF. However, research with both systems has stopped short of characterizing control of both the wrist and finger flexion DOFs simultaneously as well as the impact of biomechanical coupling on FES control.

Here, using a monkey model due to the close physiology with humans, we aim to evaluate how the established brain-controlled FES methods, using predetermined stimulation patterns for each joint independently mapped to BCI-decoded movements in the same DOFs, could extend to restoring simultaneous movements of two biomechanically coupled joints, the wrist and fingers. We placed particular emphasis on non-prehensile movements, aiming to achieve graded movements which could generalize to movements needed for functional tasks. To this end, first as a method to control this stimulation, we adapt our high-performance finger BMI [24] to decode intended finger joint angle and wrist joint angle in a proof-of-concept virtual task. We show that a linear 2-DOF BMI works well, and while the BMI control efficacy decreases after blocking sensory afferents, we verify that performance can be recovered with an additional intention estimation training step. Then we define predetermined patterns of stimulation that map finger joint angle or wrist joint angle to stimulation parameters on multiple electrodes, such that stimulation could be used to acquire BMI decoded intended movements. We show that stimulation determined by each pattern can evoke movements in a large range for the intended DOF; however, 2-DOF movements are impacted by coupling between the DOF. We lastly evaluate whether the stimulation can be continuously controlled, with a simple closed-loop strategy, to achieve target finger and wrist flexion movements simultaneously.

## Materials and methods

All procedures were approved by the University of Michigan Animal Care and Use Committee.

### Implants

**Fig. 1:**
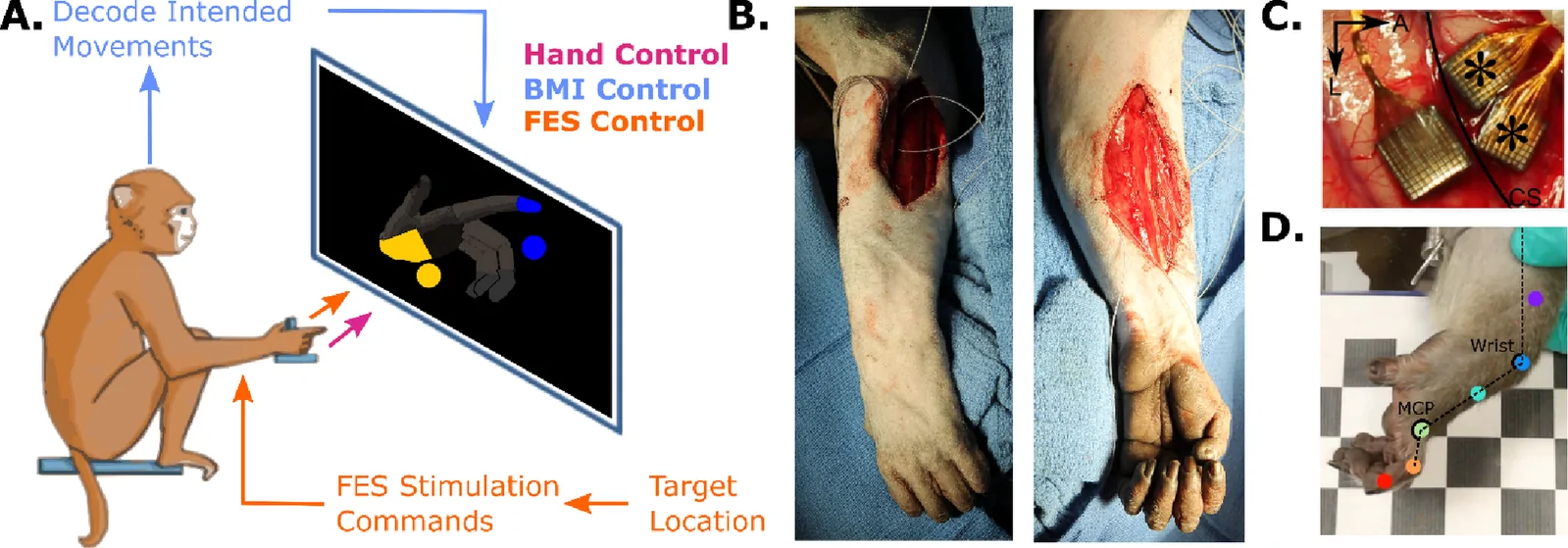
Experimental setup. **A** Illustration of the three different types of closed-loop experiments performed: hand control, BMI control, and FES control. **B** Intraoperative images from intramuscular electrode implantation in Monkey R. Electrodes were implanted in flexor and extensor muscles via either the dorsal (left) or volar (right) surgical incision and then tunneled across the shoulder to a percutaneous exit site on the back. **C** Location of Utah array implants in Monkey N. The two arrays marked with (*) are anterior to the central sulcus, in the primary motor cortex, and were used in this study (CS – central sulcus, A – anterior, L – lateral). **D** Example angle measurements using markers identified with Deeplabcut [39, 40]. Example wrist flexion and finger flexion (MCP - metacarpophalangeal) angles are marked

Two male Rhesus Macaques (Monkey N and Monkey R) were implanted with chronic bipolar intramuscular electrodes (Synapse Biomedical, Inc, Oberlin, OH, USA). Muscles were accessed via dorsal and ventral incisions on the left forearm, as described previously [22, 24, 29]. Example surgical sites are shown in Fig. 1B. In Monkey N, specific muscles were surgically identified, and implant locations were selected based on intraoperative stimulation. With Monkey R, specific muscles were surgically identified and nerve entry points to the muscles were identified. Nerve entry points were targeted for implants and hand function was confirmed with intraoperative stimulation. Bipolar electrodes were inserted and sutured into the muscle belly at the target location and then tunneled to an interscapular exit site. Monkey N was implanted with eight bipolar electrodes and Monkey R was implanted with 16 bipolar electrodes. Muscles targeted and evoked movements from intraoperative stimulation are described in Additional Table 1. Electrodes are labeled based on the muscle that they are implanted in and their expected function. Monkey N had been previously implanted (22 months prior to FES electrode implant) with two 64 channel Utah microelectrode arrays (Blackrock Neurotech, Salt Lake City, UT, USA) in the right hemisphere, targeting hand area of precentral gyrus (Fig. 1C), as described previously [36]. Notably, these arrays were implanted 1577 days before the first BMI experiments presented here.

### Experimental setup

To test FES evoked range of motion and interactions between the two DOFs, stimulation was delivered through implanted intramuscular electrodes and evoked hand movements were recorded with video. All stimulation was delivered via a networked neuroprosthesis (NNP) evaluation system [29, 37, 38] with the monkey’s hand temporarily paralyzed via nerve block (see Nerve Block). This system contained the same circuitry as the NNP used in humans (Clinical Trial NCT02329652); however, it was in a form more conducive to preclinical experimentation. The NNP system consisted of 1 power module and 3 pulse generator boards, each with 4 output channels. The return electrode of each bipolar pair connected to the same pulse generator board were electrically tied together to make one common return for each pulse generator board.

To test closed-loop continuous control, for example with FES or with the BMI, both monkeys performed a virtual target acquisition task (Fig. 1A). During the virtual target task, hand movements recorded by bend sensors were mirrored in a virtual hand in front of the monkey and juice was rewarded for moving the virtual hand to target wrist and finger flexion. Both monkeys did this task with closed-loop FES control in which computer-controlled stimulation moved the monkey’s nerve blocked hand in the manipulandum to acquire the targets with the virtual hand. Alternatively, during BMI experiments Monkey N did the virtual target task with their able-bodied hand (hand control in Fig. 1A) to acquire training data to train BMI models. Monkey N then did the virtual task with BMI control in which the monkey’s movement intentions decoded from neural activity moved the virtual hand (rather than tracked physical hand movements). BMI control was done both with and without nerve block. We used a computer running xPC Target version 2012b (Mathworks) to coordinate the experiment in real time, storing task parameters, neural data, and hand kinematics while coordinating target presentation, executing the decoder model to decode finger and wrist positions, and sending stimulation commands to the NNP as necessary.

### FES patterns

All stimulation was current-controlled and delivered at 32 ms inter-pulse intervals with either 5 mA or 10 mA amplitudes. Stimulation used charge-balanced biphasic pulses with a 50 microsecond intrapulse period. Stimulation intensity was modulated by changing the width of the initial cathodic pulse, varying between 0 and 255 microseconds. Pulse width modulation was used due to the history of successful upper limb stimulation using it [41] and the potential to evoke the same movements with a lower amount of delivered charge [42]. Stimulation patterns mapped stimulation amplitude and pulse width on multiple electrodes to a desired flexion for one DOF. Commands on these patterns ranged from 0% flexion (i.e., extension) to 100% flexion with 50% indicating a neutral posture. As such, each pattern included stimulation parameters for both flexor and extensor muscle electrodes. Example stimulation patterns for Monkey R can be found in Supplemental Figs. 1 A & 1B.

Patterns were manually designed using anatomical knowledge and results from single electrode stimulation characterization. Initial patterns were made by setting the pulse width at command extrema (0% or 100% for extension and flexion respectively) to the pulse width that evoked the largest movement in the desired DOF and direction without evoking substantial movements in the other DOF. We set the pulse widths at the 50% command (center/neutral position) to the pulse width that caused the first movement in the desired DOF, to maintain a minimal level of stiffness at center, and then the pulse width was set to 0 for movements in the direction opposite of the muscle’s contraction. The patterns then smoothly transitioned pulse widths between these command values.

At the beginning of experiments, these established stimulation patterns were tested and then adjusted if necessary. This verification was a manual process in which stimulation at the extrema were tested and increased or decreased if necessary and the stimulation at pattern center was also tested and overlap between flexors and extensors was adjusted to better achieve the center posture if necessary. As a result, patterns typically changed with each experiment (example patterns are provided in Supplemental Fig. 2).

### FES range of motion experiments

To investigate the range of movements that could be evoked in each DOF (wrist and finger flexion) with FES, as well as the impact of biomechanical coupling on movements evoked on both DOF, stimulation evoked movements were recorded on video using a Canon Eos Rebel T3 SLR camera (Canon, Melville, NY). To control the stimulation, 1 stimulation pattern, defining stimulation on multiple electrodes to extend and flex the joint, was defined for each DOF controlled (see FES Patterns). Then stimulation was tested: along individual patterns (4 experiments with Monkey R, 5 with Monkey N), on the finger pattern with wrist muscle stimulation to maintain a wrist posture (3 experiments with each monkey), and at randomly sampled command values along both patterns (2 experiments with Monkey R and 4 with Monkey N). Stimulation in these first two types of experiments was controlled by the experimenter and then stimulation at randomly sampled commands on the patterns was coordinated using xPC.

In experiments testing the finger pattern stimulation with simultaneous stimulation maintaining wrist posture, the wrist stimulation was chosen by the experimenter, through test stimulation, to be sufficient to move the wrist to the desired posture on that day but not large enough that stimulation would evoke finger movements as well. In random stimulation command sampling experiments, the random sampling by xPC selected one command value for each pattern by sampling first a separation between the two commands (to ensure the wrist and fingers were often commanded to move in different directions) and then a midpoint that the commands were centered about. Stimulation was changed to the new command values in one step and held so that static postures could be analyzed. During stimulation controlled by the experimenter the length of stimulation hold times varied (typically 2–3 s). Stimulation during the xPC-controlled random sampling occurred for three seconds before changing to a new stimulation command. All stimulation was done with the monkey’s hand temporarily paralyzed by nerve block.

### Closed-loop FES experiments

To investigate whether 2-DOF FES could be continuously controlled to dynamically acquire and hold desired target postures, computer-controlled FES and a simple feedback control algorithm were used to move the monkey’s nerve blocked hand to perform the virtual target acquisition task, continuously acquiring target finger and wrist flexion throughout the range of motion. After nerve block, flex sensors (FS-L-0073-103-ST, Spectra Symbol, Salt Lake City, UT, USA) were taped to the back of the wrist and index finger (Supplemental Fig. 3B). These flex sensors connected to a custom analog to digital converter circuit which sent serial input to the xPC system. The xPC system then displayed the measured finger and wrist flexion with a virtual hand and determined target acquisition by comparing virtual hand position to the virtual targets.

Before closed-loop tests began, stimulation patterns for both the wrist and the fingers were tested and adjusted if necessary. Flex sensor measurements were calibrated so that the virtual hand was fully flexed or extended at approximately the range of motion available from stimulation. Target presentation and stimulation were controlled by the xPC system. Target amounts of flexion for both the wrist and the fingers were selected and then targets were presented on the screen. While targets were presented to the monkey, this experiment was done under nerve block and with computer-controlled stimulation, so target visualization was not essential. The computer-controlled stimulation was ramped in the direction (flexion or extension) required to move that DOF to the target. This was done with a simple algorithm that, every 32 ms, checked if the fingers and the wrist were in their targets, and if not would update the stimulation pulse width for the joint not in the target. The update moved the stimulation by 0.39% (one command value out of the 8-bit encoded command range) on the corresponding joints stimulation pattern in the direction needed to get to the target (i.e., flexion or extension). For a trial to be successful, both the fingers and the wrist had to remain in their respective targets for 500 ms. Trials resulted in failure if this took longer than 10 s. Target sizes were 22.5% and 24.9% of the movement range for the fingers and wrist respectively.

Closed-loop FES was tested on 2 days for each monkey for a total of 164 trials with Monkey N and 278 trials with Monkey R. Targets were presented in a reduced range of movement due to muscle fatigue consisting of the middle 50% of each DOF. Targets for the wrist and fingers were picked such that the target centers were separated by up to the maximal 50% of range of movement (e.g., fingers flex, wrist extend). This was done by sampling a random separation from a uniform distribution and then a random target midpoint (about which the targets are separated) from a uniform distribution constrained to result in target centers within the middle 50% of each DOF given the chosen separation. In one experiment with Monkey R, each target was instead chosen independently to have a target center sampled from a uniform distribution of the middle 50% of the range of movement.

### BMI experiments

To investigate whether Monkey N could use a BMI to produce continuous 2-DOF movement commands, the monkey performed the virtual target acquisition task with BMI control both before and after temporary paralysis. We implemented a position/velocity recalibrated intention-trained (ReFIT) Kalman Filter (RKF) like what we have previously published with 2 finger-groups [22, 24], but with updated parameters to use the wrist instead of a second finger-group. While these experiments were performed with Monkey N, they were separated in time from FES due to a decline in brain signal quality. Decoder training involved a multistep process, as illustrated in Fig. 3A.

First, Monkey N completed at least 375 trials (average 480 trials per session where one trial is acquiring one target) of the virtual target task with hand control (hand control in Fig. 3A) in an adapted finger and wrist manipulandum (Supplemental Fig. 3B), while spiking band power [36] and finger and wrist movements were recorded. Finger movements were tracked with potentiometers in the manipulandum (RH24PC, P3 America, Leander, TX, USA) with the potentiometers connected to an Arduino Uno (Arduino, Somerville, MA, USA), which digitized the analog voltages and sent serial input to the xPC system.

Using the neural and hand movement data from hand control, a position/velocity Kalman filter (KF) was trained and then used in BMI control, in which velocity decoded from brain activity using the KF controlled the virtual hand, for at least 200 trials (average 299 trials per session). This KF BMI control data was then used as training data for the intention estimation and recalibration step for the RKF. The RKF model was then used in BMI control for at least 150 trials (average 192 trials per session) to get an initial performance, after which the nerve block was performed. Once hand function was confirmed to be lost, the same RKF model was used in BMI control again for at least 150 trials (average 186 trials per session). One more RKF model (Re-ReFIT model, ReRKF) was then trained using these post-nerve block RKF BMI control trials. The ReRKF model was then tested in BMI control for at least 125 trials (average 154 trials per session). Trials were performed consecutively with breaks only occurring between control paradigms for model training or performing the nerve block.

Targets were presented in a center-out fashion requiring flexing or extending the fingers or wrist from rest, for a total of 8 movement combinations (3 possible movements – flex, extend, or rest – on each of 2 DOF), and to 3 different magnitudes of movement (20%, 30%, and 40% of calibrated range) from center (Supplemental Fig. 4). The subsequent trial then required moving back to center. Additionally, the trial after a failed trial required moving back to center. Targets presented had a diameter of 16.9 to 18.8% of the movement range for the finger target and 18.7 to 20.8% of the movement range for the wrist target. The wrist target was made wider to accommodate a larger wrist visualization as the visualization size resulted in a larger range where the visualization appeared to be within the target. Targets had to be held for 500 ms in BMI control and 750 ms in hand control. In one run of hand control trials, the target hold time was mistakenly set to 500 ms instead of 750 ms. Trials were considered failed if targets were not reached and held within 10 s.

### Extracting joint angles from video

During FES range of motion experiments, wrist and index MCP angle were extracted from video of the hand. To ensure consistent video, the camera was mounted on a tripod and angled such that the forearm just proximal to the wrist was at the edge of the frame. The monkey’s forearm was braced by an experimenter to keep the hand off the table and moving within the plane of the recorded video. Deeplabcut (DLC) models [39, 40] were trained to extract hand poses from the video. To train models, 20 frames were extracted from up to three videos in an experiment. Frames were manually labeled using the DLC API and then the network model was trained.

This work used the provided ResNet-50 model and initiated network training with the provided pre-trained weights. Seven locations were labeled in each frame: the forearm, wrist, back of the hand, index metacarpophalangeal (MCP) joint, index proximal interphalangeal (PIP) joint, and index distal interphalangeal (DIP) joint. Due to inconsistencies in recorded video each day, this process was done for each experiment where continuous joint angles were extracted. After evaluating models on videos, frames were filtered out if model certainty was not above 95% for at least two of the labels in the frame.

Wrist and index MCP angles were extracted from DLC labels using a custom MATLAB script. An example frame with wrist and MCP angles labeled is shown in Fig. 1D. The wrist angle was defined as the angle between a vector from the forearm to the wrist label and a vector from the wrist label to the MCP label. Due to commonly occurring obstructions of the forearm marker (i.e., the edge of the frame, the arm restraint, or the experimenters), the edge of the frame at the forearm was used instead of the forearm marker. The marker location was chosen such that the vector from the forearm to the wrist was perpendicular to the edge of the frame. The index MCP angle was defined as the angle between a vector from the wrist label to the MCP label and a vector from the MCP label to the PIP label. Extracted angles were then smoothed with a Gaussian-weighted moving average filter with a 0.5 s window. In range of motion tests where continuous joint angles were not needed, single frames were extracted from videos and joint angles were measured using ImageJ (version 2.35).

### Nerve block

To achieve temporary hand and wrist paralysis, we performed an ultrasound guided nerve block of the median, radial, and ulnar nerves just proximal to the elbow prior to stimulation. First, lidocaine (2%) was injected subcutaneously near the planned injection sites to help prevent discomfort from injections. Then a solution of lidocaine (2%) and epinephrine (1:100 000) was injected into the perineural space surrounding the nerve under ultrasound guidance. In later experiments, the lidocaine dosing was reduced to 1% without a noticeable change in the efficacy of nerve block. Typical blocks involved injecting 1.0 mL to 1.5 mL around each of the three nerves for a total lidocaine injection of up to 8 mg/kg at 2% lidocaine, or 4 mg/kg at 1% lidocaine. Note that this maximum lidocaine dosing is smaller than similar studies which have used approximately 20 to 30 mg/kg [26, 27]. This blocked hand function for sufficient time to perform FES tests (about two hours) without re-dosing. Block effectiveness was tested with a grasping task where we observed their motor capabilities while they attempted to grasp offered treats.

### Neural features

Motor-related spiking band power (SBP) [36] was recorded from 96 channels of Monkey N’s Utah arrays in primary motor cortex. 96 channels were used due to available recording hardware. Briefly, we configured the Cerebus Neural Signal Processor (Blackrock Neurotech) to sample signals at 2000 Hz and band-pass filter the recorded signals between 300 and 1000 Hz (second order Butterworth filters). Continuous neural data was sent to the xPC computer which calculated a mean absolute value in non-overlapping 32-ms bins for decoding. Neural channels were masked to only use channels that contained morphological spikes during the experiment or in the recent past and that did not have excess noise, resulting in an average of 80.7 channels per session.

### BMI decoder training

Decoder training matches training for a KF for 2 finger groups [24], but with updated parameters to use the wrist instead of a second finger group. The KF and RKF assume a kinematic state with one position and velocity for each degree of freedom. For the 2D finger and wrist task, there is one position and velocity for the wrist and one position and velocity for the fingers:

$$\:{\mathrm{x}}_{\mathrm{t}}=\left[\begin{array}{c}{\mathrm{P}}_{\mathrm{I}}\\\:\begin{array}{c}{\mathrm{P}}_{\mathrm{W}}\\\:{\mathrm{V}}_{\mathrm{I}}\\\:\begin{array}{c}{\mathrm{V}}_{\mathrm{W}}\\\:1\end{array}\end{array}\end{array}\right]$$

Where $\:{\mathrm{x}}_{\mathrm{t}}$ is the kinematic state at time t, $\:{\:\mathrm{P}}_{\mathrm{I}}$ and $\:{\mathrm{V}}_{\mathrm{I}}$are position and velocity for the index finger, and $\:{\mathrm{P}}_{\mathrm{W}}$ and $\:{\mathrm{V}}_{\mathrm{W}}$ are position and velocity for the wrist. Note that the fingers were grouped together in visualizations by giving the rest of the fingers the same kinematics as the index finger. The KF updated the kinematic state at 32 ms intervals by combining a prediction of the state given the previous state with a state update gained from the observation model which relates neural activity to the kinematic state. The positions displayed with the virtual hand were computed by integrating the decoded velocities and adding the result to the previous positions.

The RKF and ReRKF models followed the same KF algorithm but used retrained model parameters. To train these models, the decoded velocities from the BMI trials used as training data were augmented by identifying time steps with velocities in a direction away from the target and rotating the velocities to point towards the correct 2D target vector but not rescaling the vector (as illustrated in Supplemental Fig. 5). This matches the assumption that the monkey intended to be moving towards the target during closed-loop trials. New model parameters were then trained using the augmented velocities. During use of the RKF and ReRKF models, there was no further modification of the velocities.

### Closed-loop performance metrics

We measured performance during FES closed-loop trials and BMI trials with success rate and acquisition time. In FES control, blocks of trials were excluded if both DOF started in their target, or if flex sensors were out of place. Presented results used consecutive trials without excluded trials. Success rate was then measured as the ratio of successful FES or BMI trials to the number of valid FES or BMI trials. Acquisition time was measured as the time required to complete the trial, minus the hold time and was only measured for valid successful trials. In BMI trials, we also calculated an orbiting time, that is the time between first reaching both targets simultaneously, and successfully holding the targets, minus the hold time. Orbiting time was measured for all valid and successful trials with a non-zero orbiting time.

### Range of motion analyses

Finger and wrist ranges of motion are calculated as the difference in index MCP joint flexion or wrist joint flexion, respectively, between a frame when stimulation is flexing the fingers or wrist and when stimulation is extending the fingers or wrist. One finger range and one wrist range was obtained for both the wrist and finger pattern tested on one experimental day. When testing the 2-DOF movement ranges, evoked movements are calibrated to the maximum angles that an individually tested pattern moved the joint to. For example, a wrist extension of 1.0 would be equivalent to the angle that the wrist pattern extended the wrist to on that day, whereas a finger extension of 1.0 would be equivalent to the angle that the finger pattern extended to fingers to. In one experiment with Monkey N the wrist pattern was not tested initially, and the wrist movements are calibrated to the movements due to the finger pattern. Calibrated angles are then plotted into the two-DOF wrist and finger extension space. A boundary is calculated around all angles using the MATLAB boundary function with a shrink factor of 0.5 which balances finding a convex hull boundary and a compact boundary around the set of angles.

When calculating the goodness of fit between stimulation and the evoked postures during the random stimulation sampling, one wrist and finger posture was calculated per stimulation by averaging the posture in the last 0.5 s of the three second stimulation. A linear regression, which included a bias term, was then fit between the stimulation command and the evoked posture. A separate model was fit between each stimulation command, or both stimulation commands, and either the wrist or finger posture and then a coefficient of determination was calculated. A bootstrap method was used to estimate a variance on the coefficient of determination. Each model fitting was performed 100 times using n stimulation trials sampled with replacement, where n was the number of three second stimulation trials performed.

### Statistical analyses

Unless otherwise noted, significance for metrics such as evoked ranges of motion, and bootstrapped coefficients of determination, were tested with two-sample t-tests. Significant changes in success rate between two runs of trials was determined using a chi-squared test and significant changes in median BMI performance metrics was determined used two-sample Kolmogorov-Smirnov tests. Significant changes in BMI performance before and after nerve block across multiple sessions is tested with a two-way ANOVA where one factor is trial type (i.e., before block RKF, after block RKF, or ReRKF) and a second factor is experiment day.

## Results

### BMI control of virtual finger and wrist movements

**Fig. 2:**
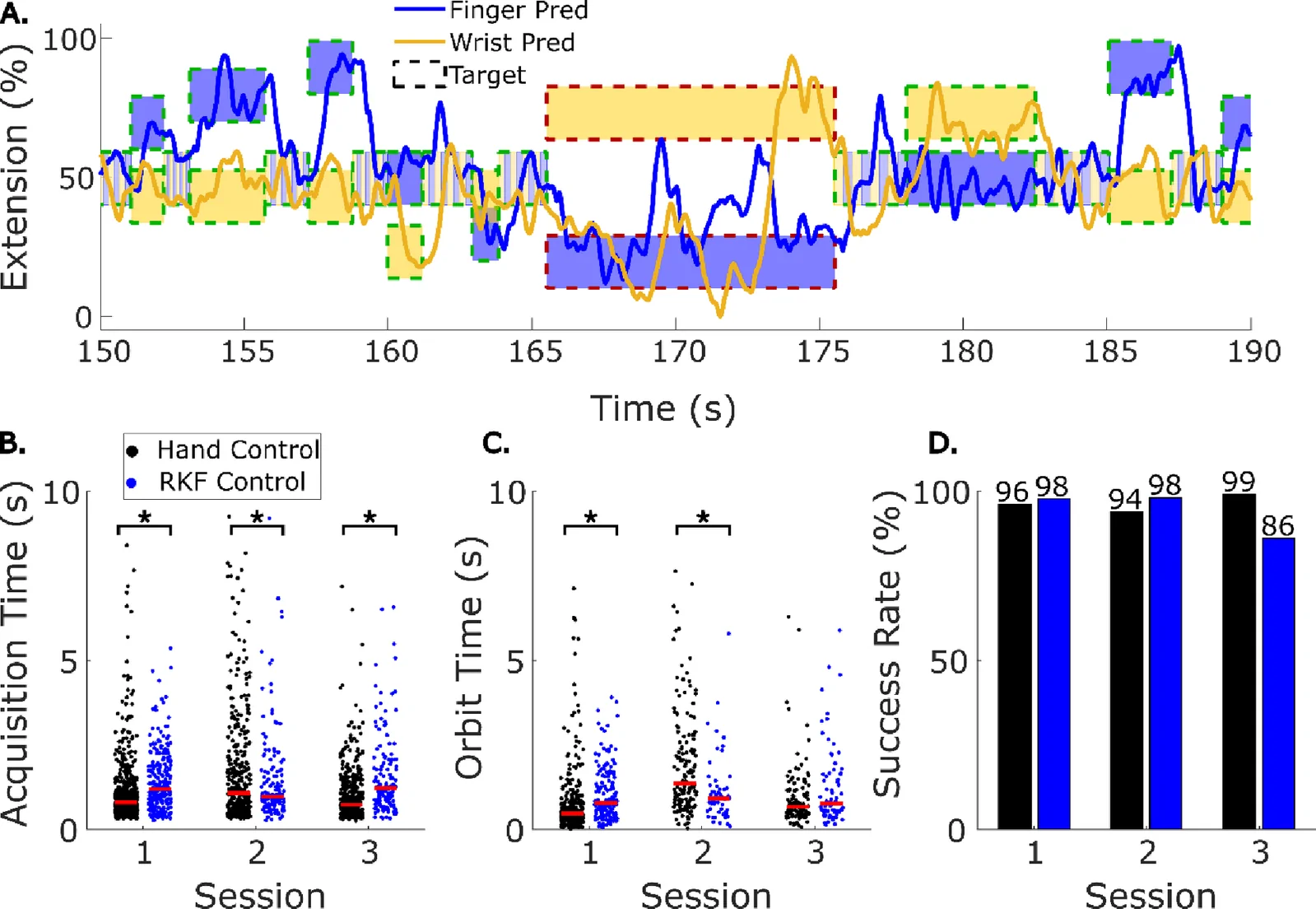
Two-DOF BMI control of virtual wrist and fingers. **A** Example decoded finger and wrist extension from a closed-loop BMI experiment. Green target boundary indicates trial success, and red target boundary indicates failure. **B** Distributions of acquisition time for all 3 sessions with RKF control in blue and hand control in black. Each dot indicates an individual successful trial. Red bars indicate the median. Significance level was *p* < 0.05 using two-sample Kolmogorov-Smirnov test with Bonferroni correction. **C** Distribution of orbit times for all three sessions, following the format in (**B**). **D** Success rate using hand control (black) or BMI control (blue) during each session

We first asked how well a BMI can generate a command signal from brain activity to ultimately control 2-DOF FES aimed at restoring coordinated finger and wrist flexion movements. We trained a recalibrated feedback intention-trained (ReFIT) Kalman filter model (RKF), following the procedure we have used previously [22, 24], enabling Monkey N to control finger and wrist flexion of a virtual hand simultaneously with only the neural activity from implanted Utah arrays (Fig. 1A, BMI control). Figure 2A shows example decoded finger and wrist flexion trajectories from a closed-loop BMI session with Monkey N using the RKF model (also see Additional Movie 1). We tested BMI control of wrist and finger flexion on 3 experimental days. Performance was measured with acquisition time, orbit time, and success rate (Fig. 2B-D). Performance with the RKF model was comparable to that of using the hand to control the manipulandum with a similar success rate across sessions (96.5% down to 94.5%, *p* = 0.041 chi-squared test), only 1 session with a higher median orbit time (311.5 ms increase, *p* < 6e-8 two-sample Kolmogorov-Smirnov test), and 2 sessions showing increases in median acquisition time (391 ms and 489 ms increases, *p* < 5e-11, two-sample Kolmogorov-Smirnov test).

**Fig. 3:**
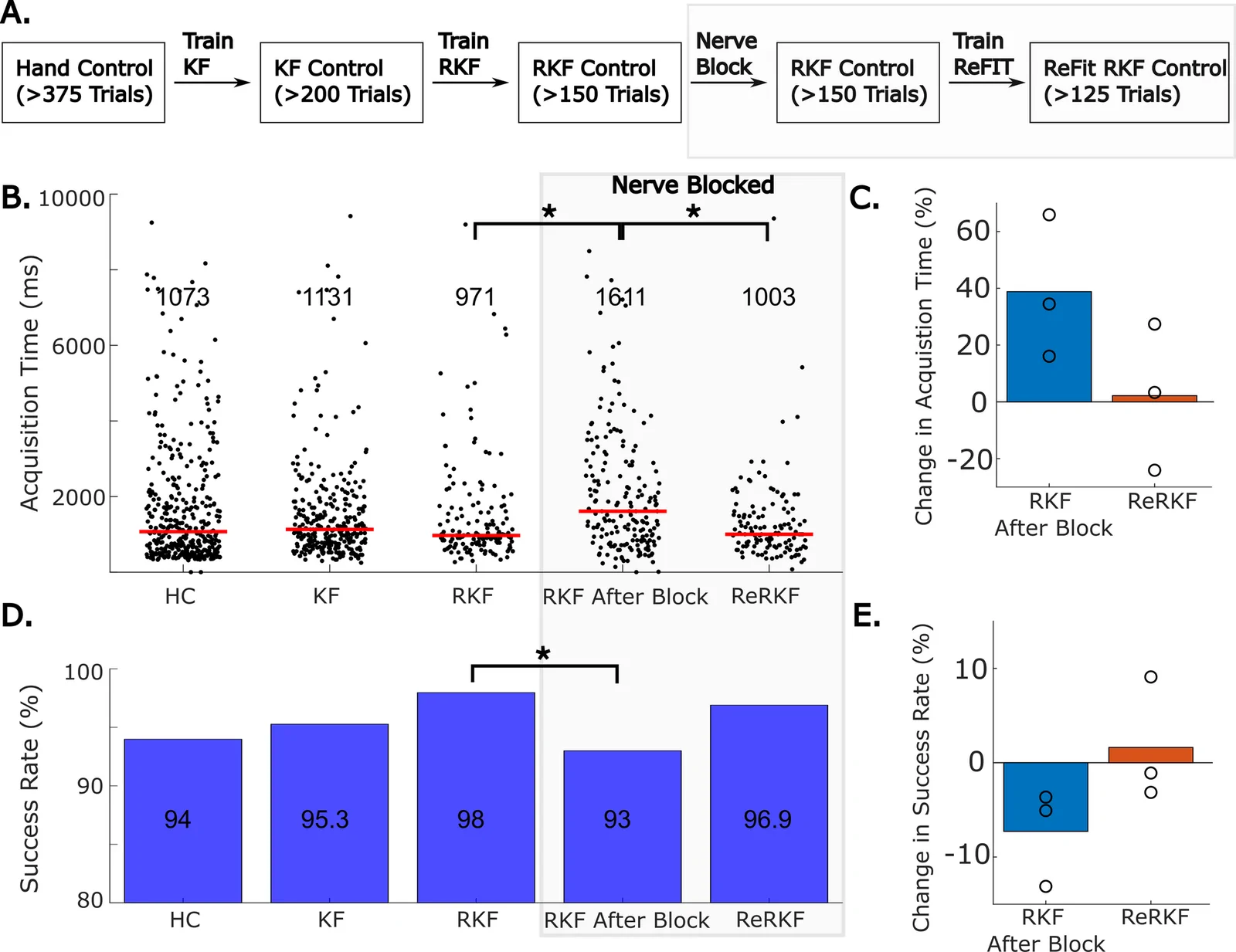
Recovering BMI performance after nerve block. **A** Illustration of the model training process with respect to the nerve block. **B** Acquisition times during an example session. ReRKF is the decoder that was trained in a second ReFIT step after nerve block. Red lines and numbers near the top of each column indicate the median acquisition time for that set of trials. Significance stars indicate *p* < 0.05 two-sample Kolmogorov-Smirnov test with Bonferroni correction. **C** Change in median acquisition time during three experiments where an RKF was tested before and after nerve block and then compared to the ReRKF. The change is measured with respect to RKF performance before nerve block. **D** Success rates during an example session. Significance indicates *p* < 0.05 with chi-squared test. **E** Percent change in success rate during the 3 experiments where RKF was tested before and after nerve block and compared to ReRKF. Change is measured with respect to RKF performance before nerve block

For translational purposes, it will be important to decode intended movements in a patient with spinal cord injury whereas our monkey model is able-bodied. To this end, we tested whether the trained Kalman filter could still decode 2-DOF of intended movements in a closed-loop BMI task after the same nerve block used in FES experiments, which eliminates movement and proprioception similar to a spinal cord injury. First, an RKF model was trained and tested prior to the nerve block. Then the nerve block was performed and the same RKF model was tested again (Fig. 3A). Figure 3B and D show average acquisition time and success rate respectively during each set of trials in one example session. In this example session, BMI performance decreased while using the same RKF model after nerve block, median acquisition time increased from 971 ms to 1611 ms (*p* < 1e-5, two-sample Kolmogorov-Smirnov test), and success rate decreased from 98% to 93% (*p* = 0.03, chi-squared test).

Interestingly, some performance was regained by applying a second ReFIT calibration stage using the successful trials from BMI control after nerve block. In the example session, using the retrained RKF (ReRKF) improved median acquisition time to 1003 ms (from 1611 ms; *p* < 4e-5, two-sample Kolmogorov-Smirnov test) and success rate to 96.9% (from 93%; *p* = 0.13, chi-squared test). We repeated the BMI task in three nerve block experiments. First, when testing the same RKF before and after nerve block, BMI performance consistently decreased after the nerve block. Across these 3 days, acquisition time increased by an average of 38.8% (*p* < 1e-9, two-way ANOVA) (Fig. 3(c)) and success rate decreased by 7.2% (*p* < 1e-4, chi-squared test) (Fig. 3(e)). Then, retraining the RKF using ReFIT on BMI trials after the nerve block successfully regained performance in all 3 experiments. ReRKF performance showed equivalent acquisition times (*p* = 0.32, two-way ANOVA) (Fig. 3) and success rates (*p* = 0.68, chi-squared test) (Fig. 3E) compared to before block RKF performance. Performance metrics for each experimental session are given in Additional Table 2. Notably, the success rates and acquisition times in the first two sessions were not significantly different between hand control trials and the ReRKF trials (*p* > 0.03 two-sample Kolmogorov-Smirnov test). Ultimately, this shows that a linear Kalman filter can decode intended finger and wrist movements well after nerve block as long as a retraining step is used to account for the loss of sensory information.

### Evoked movements with stimulation targeting 1-DOF

We next asked how well coordinated stimulation of multiple intramuscular FES electrodes could move the wrist and finger DOFs individually, with the goal that selective movements could easily be combined into 2-DOF movements. Following conventional FES control methods [41], a stimulation pattern was defined for each of the 2-DOF (wrist or finger flexion). While stimulation patterns typically define the parameters to elicit a functional movement (e.g., grasp), we used these patterns to define stimulation parameters for both flexor and extensor muscles to move a desired DOF continuously from extension (at low commands) to flexion (at high commands) [29]. Supplemental Figs. 1A and 1B show example finger and wrist patterns respectively for Monkey R.

**Fig. 4:**
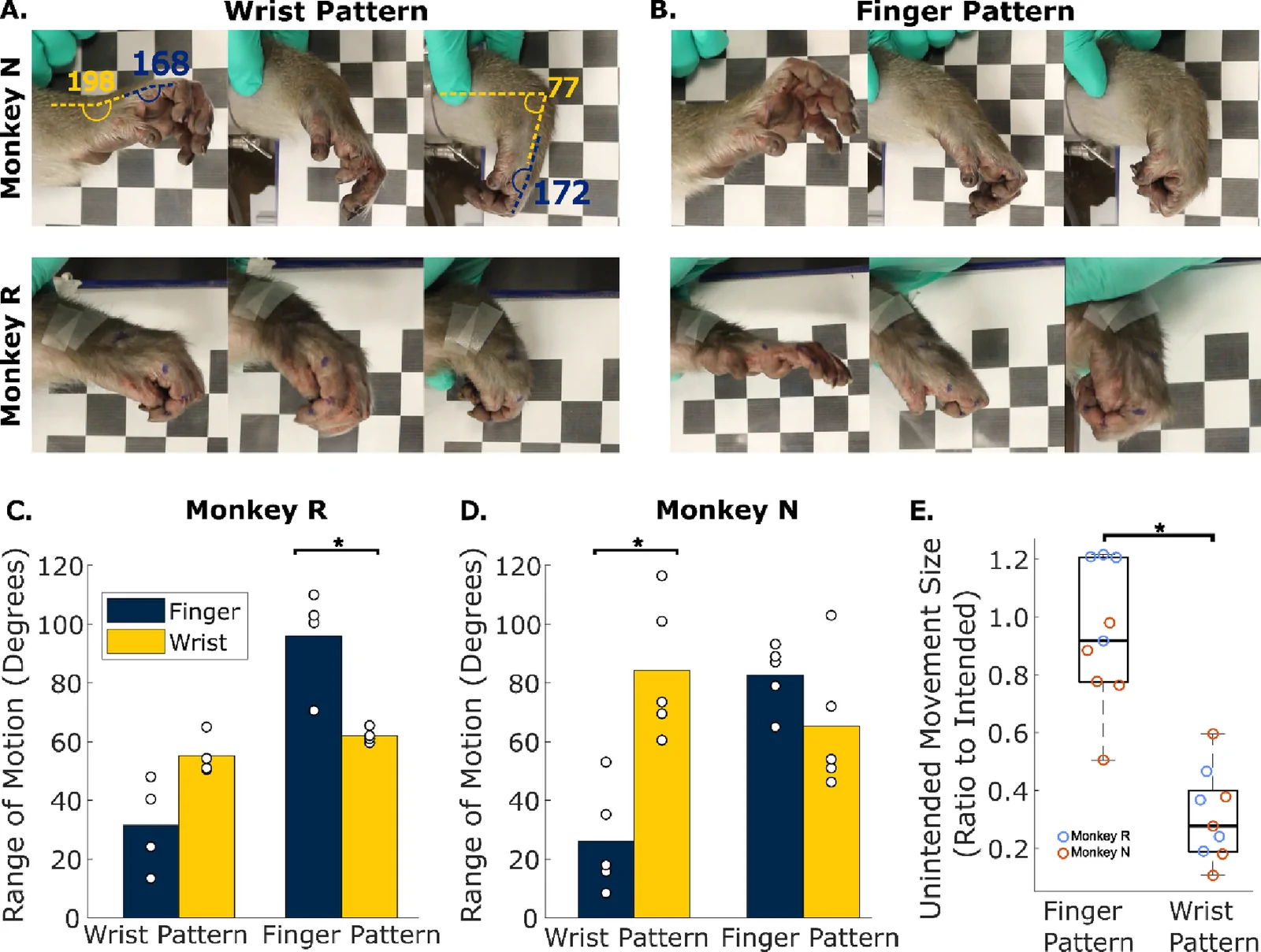
Evoked range of motion using stimulation patterns. **A** Example evoked movements from testing the wrist stimulation pattern on one example day with each monkey (top – Monkey N, bottom – Monkey R). **B** Example evoked movements from testing the finger stimulation pattern on one example day with each monkey (top – Monkey N, bottom – Monkey R). **C**, **D** The range of evoked movements in the index MCP joint (blue) and wrist joint (yellow) using the wrist pattern and finger pattern for Monkey R (**C**) and Monkey N (**D**) respectively. Open circles indicate measurements from different experiments where patterns were tested. Significance stars indicate *p* < 0.05 with paired t-test and Bonferroni correction. **E** Size of “unintended” movements, measured as the size of the movement on the opposite joint, i.e., wrist joint for finger patterns and finger joint for wrist patterns, presented as a ratio to the ROM given by the intended pattern for that joint. Open circles indicate measurements from different experiments. Results are grouped for both monkeys. Boxes indicate the 25th to 75th percentiles and the central mark indicates the median. Significance determined with two-sample t-test, *p* = 7e-6

We observed that stimulation via each pattern successfully moved the intended DOF across a large range of motion (ROM) (Fig. 4A and B). To quantify these movements, we measured the joint flexion angles for the wrist joint and the index metacarpophalangeal (MCP) joint during each evoked movement (example measurements in Supplemental Figs. 6A and 6B). The finger patterns moved the fingers through a large ROM for both monkeys (Fig. 4C and D), averaging 88.6 degrees (SD = 15.0) pooled across sessions and both monkeys. Similarly, the wrist patterns moved the wrist through an average range of 71.3 degrees (SD = 23.0). For context, studies on functional ROMs required for activities of daily living report an 80 degree functional wrist flexion range [6] and 53.3 and 52 degree MCP flexion ranges [43, 44] in human participants.

While stimulation achieved large ROMs, both patterns also evoked unintended movements in the other joints (Fig. 4E). Most notably, the finger patterns for both monkeys evoked large wrist movements, with a median ROM 91.7% the size of the wrist ROM evoked by the wrist patterns, likely due to these muscles crossing the wrist joint. Wrist patterns, however, evoked less unintended movements, moving the fingers through a median range only 27.9% the size of finger ROM evoked by finger patterns. Additionally, wrist patterns moved the wrist joint in a range 150.4% larger than they moved the fingers (*p* =1e-3 paired t-test, combined across monkeys and sessions). The relative selectivity with which wrist patterns can evoke wrist movements is promising for individuating the wrist from the fingers; for example, by using the wrist pattern to stabilize the wrist during finger movements.

### Simultaneous stimulation on both patterns can move both DOF separately

**Fig. 5:**
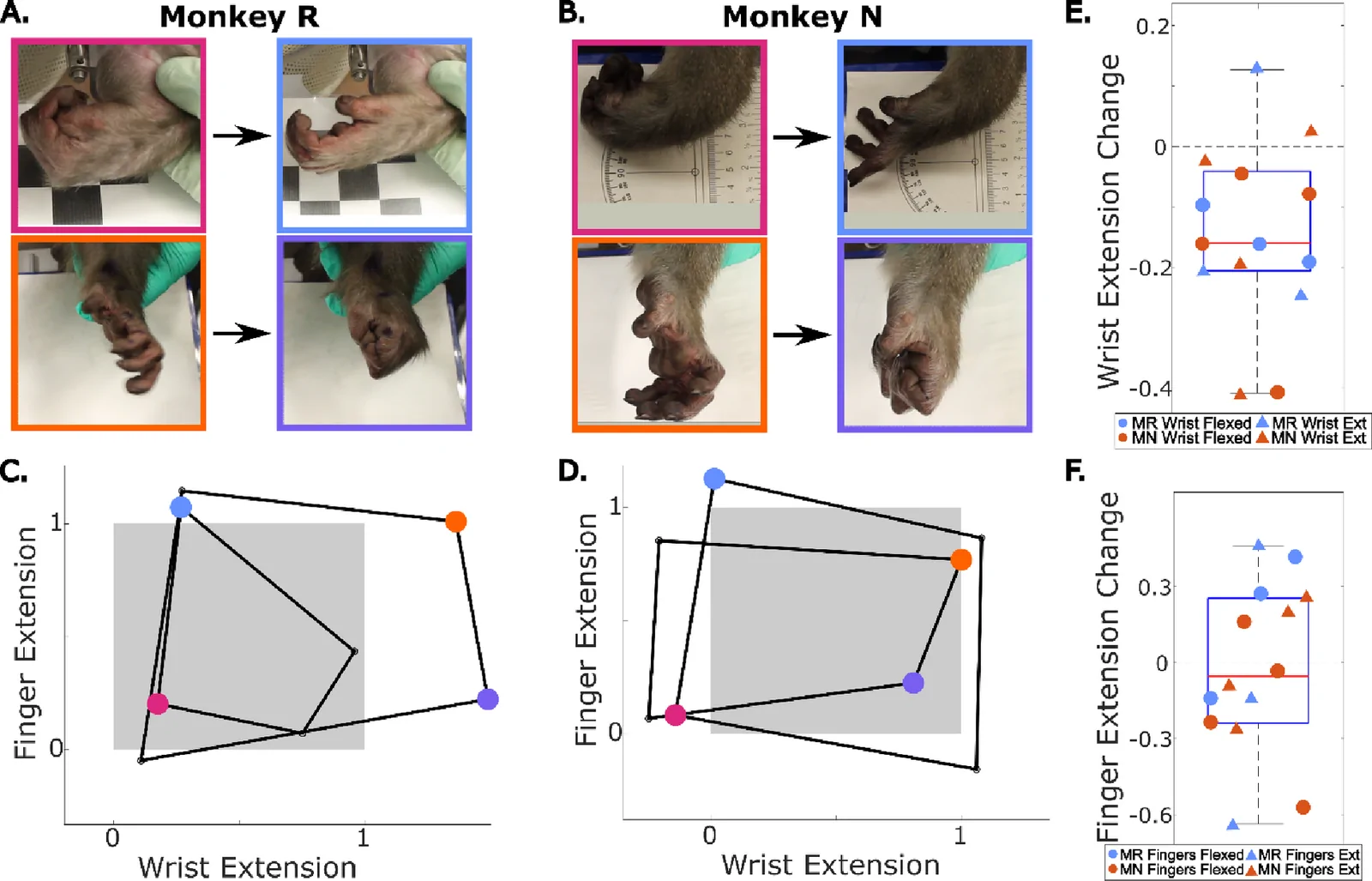
Range of motion using two-patterns simultaneously. **A** Example stimulated movements opening or closing the fingers with simultaneous wrist stimulation holding the wrist in flexion (top) or extension (bottom). Results for Monkey R. **B** Same as (**A**) but for Monkey N. **C** Measured finger and wrist angles when opening and closing the fingers with the wrist held fully extended or fully flexed by wrist muscle stimulation. Angles for postures on the same day are connected to make one quadrilateral. All four postures were tested on two days for each monkey. Colored points correspond to the frames in (**A**) with matching colored borders. Extensions are calibrated to the 1-DOF ROM available (Methods). For example, a wrist extension of 1 is the angle that the wrist pattern extended the wrist to on that day. The grey box is a unit square corresponding to the calibration. **D** Same as (**C**) but for Monkey N. **E** Change in wrist extension (fraction of calibrated ROM) when the fingers were stimulated to move to flexion while wrist stimulation actively held the wrist either flexed (circles) or extended (triangles). This was done on 4 days for Monkey N (MN - orange) and 3 days for Monkey R (MR - blue). **F** Difference in stimulation-held finger posture (extension (triangles) or flexion (circles); fraction of calibrated ROM) with stimulation extending the wrist compared to flexing the wrist

We next tested whether stimulation with the wrist and finger pattern simultaneously could successfully move the monkey’s nerve blocked wrist and fingers separately. First, we designed stimulation to move the fingers while maintaining static wrist postures, and second, we examined the attainable range of 2-DOF movements by sampling stimulation commands on both patterns. To start, wrist stimulation was used to stabilize wrist posture during stimulation to open and close fingers (Fig. 5A and B, & Additional Movie 2). We measured the finger and wrist angles during 4 evoked movements, fingers flexed or extended with wrist flexed or extended, to illustrate the 2-DOF ROM for each session (Fig. 5C and D). Evoked movements explored a large portion of the 2-DOF ROM, with a calibrated area averaging 101.8% (SD = 51.9%) of the ROM.

There are two effects due to biomechanical coupling that we expect to impact the ROM available in 2-DOF stimulation. First is active coupling, where extrinsic finger muscles exert torque on the wrist when activated. The second is passive coupling, where changes in wrist posture change the tendon length of extrinsic finger muscles making finger flexion or extension easier. We found a significant active effect as stimulation that flexed the fingers also significantly flexed the wrist, despite the wrist pattern stimulation acting to stabilize the wrist, with a median change in wrist extension of -17.5% of the movement range (*p* = 0.0015) (Fig. 5E). Alternatively, the fingers tended to be more flexed when stimulation extended the wrist, but the effect was not significant (Fig. 5F), indicating little passive effect due to wrist posture.

**Fig. 6:**
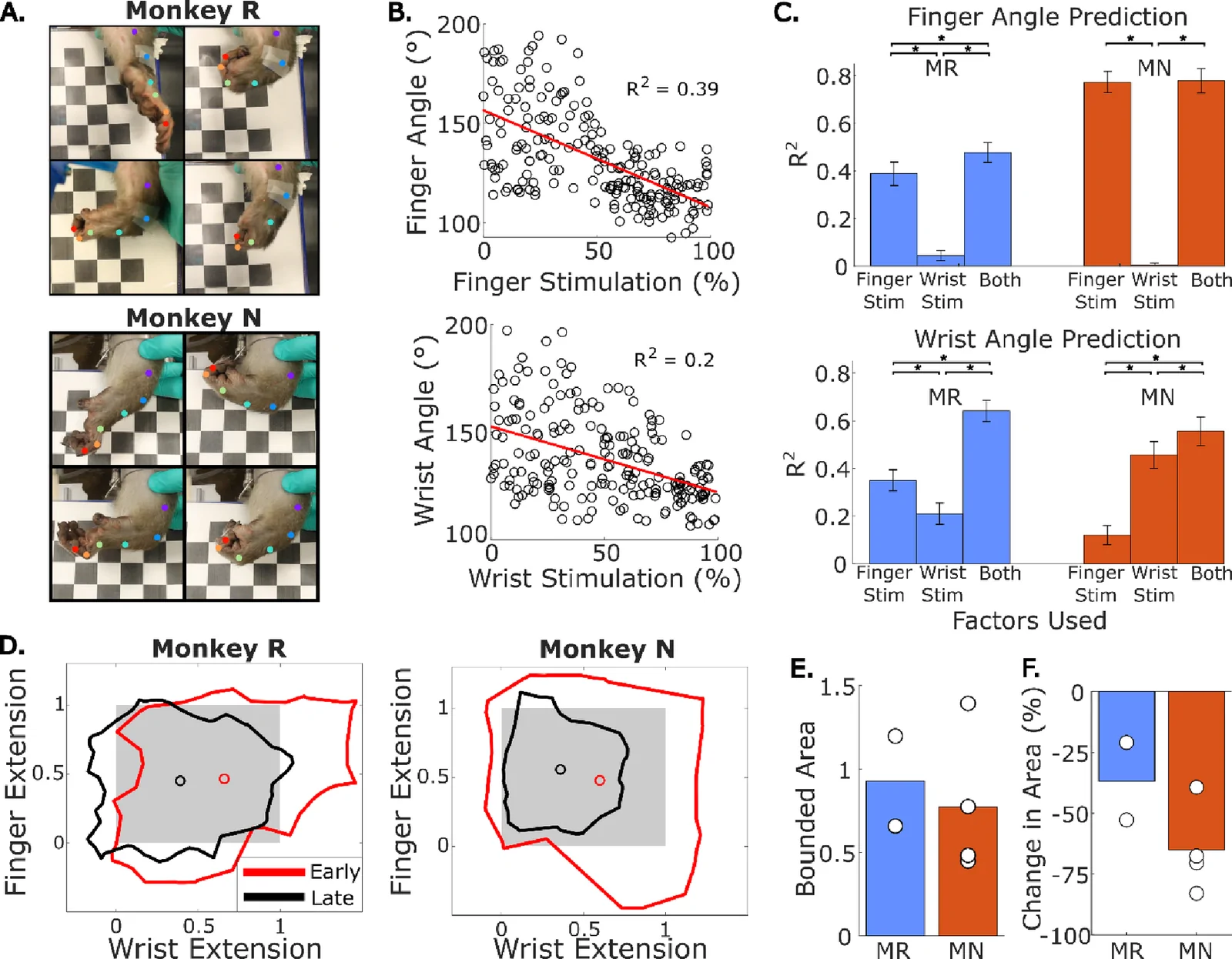
Movement range using graded stimulation on both patterns. **A** Example frames from video of 2-DOF stimulation with markers estimated using DLC for Monkey R (top) and Monkey N (Bottom). **B** Evoked posture versus stimulation command for the pattern intended to move that joint in one example session with Monkey R (top – finger, bottom – wrist). Angles are taken as the average joint angle during the last 0.5 s of the 3 s stimulation. Red lines indicate linear fits between stimulation command and joint angle. **C** Variance explained in finger angles (top) and wrist angles (bottom) by the linear fits from the factors given by the x-axis, either finger pattern stimulation command, wrist pattern stimulation command, or both. MR = Monkey R. MN = Monkey N. Results are for one example session for each monkey. Significance indicates *p* < 0.05 via two-sample t-test with Bonferroni correction. **D** Boundaries drawn containing measurements of each finger extension and wrist extension from video frames when stimulation was being administered in the first 3 min (red) or last 3 min (black). Angles are pooled from two days with Monkey R and four days with Monkey N. Angles are calibrated to the ROM available from stimulation in the respective pattern for that day. The frames at the highest and lowest 0.1% of measurements in each DOF are excluded. Grey box is a unit square corresponding to the calibration. **E** Size of bounded area from early trials. Area is calculated using calibrated movements so that values indicate a proportion of the calibrated ROM. Each dot indicates the measurement for one experimental day, there were 2 days for Monkey R and four days for Monkey N. **F** Change in bounded area between the first 3 min and last 3 min using two days for Monkey R and four days for Monkey N

Having defined the limits of the 2-DOF movement range, we next sampled combinations of stimulation commands throughout the two stimulation patterns to examine to what extent the evoked movements in each DOF were graded and independent. Figure 6A illustrates example movements for each monkey as stimulation was stepped between randomly sampled commands along the two patterns (as described in Methods). As might be expected, we found that stimulation command levels could predict the evoked postures of their intended DOF with some accuracy (Fig. 6B-C). In one example session with each monkey, finger pattern stimulation commands predicted evoked finger posture with an R^2^ of 0.39 and 0.77 for Monkey R and N respectively (Fig. 6C top), while wrist pattern stimulation commands predicted wrist posture with an R^2^ of 0.20 and 0.45 (Fig. 6C bottom). Interestingly, because of the coupling between finger muscles and the wrist, finger pattern stimulation commands also explained a significant amount of evoked wrist posture, with a R^2^ of 0.35 and 0.12 for Monkey R and Monkey N respectively in one example session for each monkey (Fig. 6C bottom). Subsequently, wrist postures were predicted better using stimulation commands from both patterns compared to only using wrist pattern stimulation commands, a 0.43 and 0.10 increase in R^2^ for Monkey R and Monkey N respectively. This effect was smaller for the finger postures which are expected to have no active effect from wrist muscles (Fig. 6C top).

In testing the 2-DOF ROM during the sampled stimulation experiments, we observed noticeable changes in the ROM over the course of each experiment, presumably due to muscle fatigue. Stimulation sessions lasted for several minutes (average 8.35 min [SD = 1.33] or 167 3-second stimulations [SD = 26.6]), and we observed a decrease in the evoked ROM over the course of the experiment. Figure 6D illustrates the evoked ROM in the first or last three minutes (approximately 60 stimulations) of an experiment for each monkey. We observed initial movements through a large portion of the finger and wrist movement space (Fig. 6E); however, in the last 3 min Monkey R’s movement area was 36.9% smaller and Monkey N’s movement area was 65.1% smaller than movements in the first 3 min (Fig. 6F). This fatigue effect was largest for wrist extension for both monkeys (Supplemental Fig. 7).

### Closed-loop control of 2-DOF FES

**Fig. 7:**
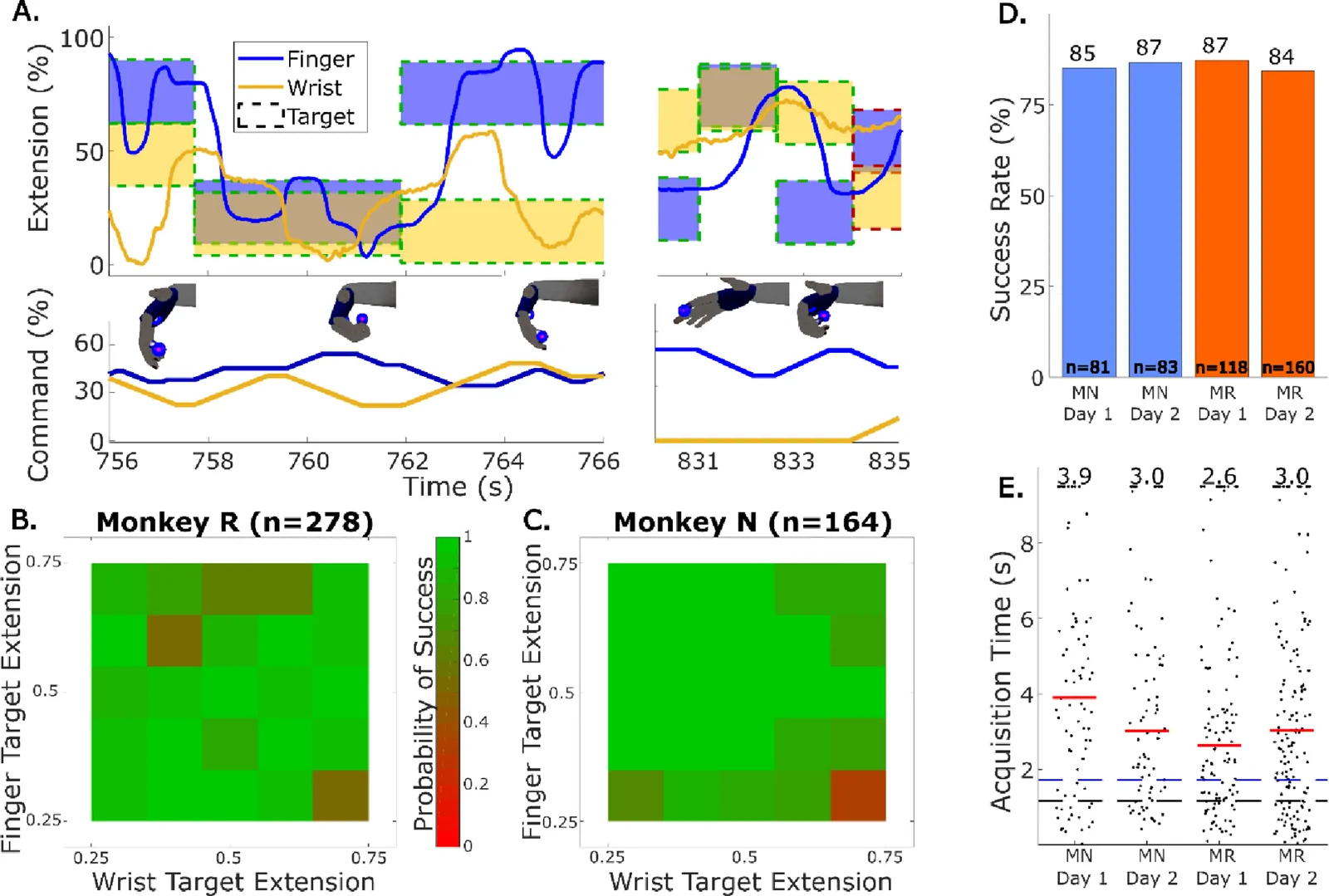
Closed-Loop Control of two-DOF FES. **A** Example finger and wrist extension (top) and FES commands (bottom) from closed-loop FES control. Blue and yellow patches indicate targets; a green border indicates success while a red border indicates failure. Representative images of the virtual hand are included to illustrate the target movement at that time. **B** Probability of closed-loop FES trial success by target location, pooled across 2 experiments for Monkey R. **C** Same as (**B**) but for Monkey N. **D** Success rate for closed-loop FES in each session, two sessions per monkey. MN = Monkey N, MR = Monkey R. **E** Acquisition times for successful trials using FES. Red bars and numbers above each column indicate the median acquisition time for that session. Dashed lines indicates an example acquisition time from Monkey N using hand control (black) or from using the ReFIT Kalman Filter (blue)

With a large range of 2-DOF wrist and finger movements available using FES, it is desirable to continuously command this stimulation to control the movement dynamics of the wrist and fingers to reach then hold postures throughout the available 2-DOF ROM. While evoked movements on the 2-DOF may not be independent, a control algorithm could compensate for this by tuning the stimulation command on each pattern to reach the target. To test this, we used a closed-loop virtual finger and wrist target acquisition task in which computer-controlled FES moved the monkey’s nerve-blocked hand to acquire target postures (see Fig. 7A for example trajectories in Monkey R and Additional Movie 3 for example virtual hand control). To enable targets to be reached at even moderate levels of fatigue, target postures were restricted to a reduced range consisting of the middle 50% of the ROM for each joint. In 2 experiments for each monkey, closed-loop control obtained 84% success or better (Fig. 7D). However, we observed difficulty completing trials with this control algorithm with both monkeys when the wrist was extended and the fingers were flexed (Fig. 7B and C, Supplemental Fig. 9), likely due to finger flexor activation pulling the wrist out of extension. While average acquisition times were slower than a representative able-bodied control acquisition time from Monkey N (Fig. 7E), the high success rates indicate that, despite a lack of independence in evoked movements, a simple control algorithm could use the 2 independent stimulation patterns to acquire 2-DOF targets in closed-loop control.

## Discussion

In recent years, BMI studies have demonstrated unprecedented success in uses such as controlling computer tablets and cursors [18, 19, 45], controlling prosthetic arms [46], and decoding intended text and speech [16, 17]. Controlling FES on independent DOF is analogous to BMI control in which independent joint angles are decoded from neural activity [25, 29]. Previous studies have decoded finger movements [24, 47], or decoded wrist movements [25]; however, it was unknown how well this would extend to simultaneous wrist and finger movements, due to the biomechanical coupling of the joints [22]. Here, we have shown that Utah microelectrode arrays implanted in the finger area of the primary motor cortex of one animal can decode simultaneous finger flexion and wrist flexion, achieving BMI performance similar to performance controlling the virtual hand with their able-bodied hand.

Additionally, we have shown that this decoding approach works after nerve block using a retraining step like methods that have become standard in several, previous BMI studies [23, 48, 49]. While not examined in this work, BMI performance is thought to decrease after nerve block due to the change in sensory input to motor cortex [29]. The presence of sensory input to motor cortex [50] also implies that in incomplete SCI, stimulation which causes sensory percepts may impact BMI performance. However, successful combinations of BMI and FES have been demonstrated in a nerve block model [29], and participants with spinal cord injury [25, 31].

While BMIs have demonstrated the ability to decode dexterous movements and continuous movements in multiple-DOF, previous upper limb FES studies have primarily only restored discrete functional grips [26, 35, 51, 52], 1-DOF continuous finger movements [29, 53], or 2-DOF continuous movements in the arm [25, 31]. Intramuscular FES is promising for restoring dexterous hand movements due to its ability to selectively activate portions of muscles and evoke selective movements. With more selective movements available, stimulating with multiple electrodes can cover a larger basis of hand movements. To this end, we evaluated how well stimulation can restore wrist and finger flexion independently to form the basis for successfully controlling continuous finger and wrist flexion simultaneously. We found that stimulation targeting either the finger flexion DOF or wrist flexion DOF successfully evokes large movements, comparable to the functional ROM for MCP and wrist joints during activities of daily living [43, 44]. However, the range of motion in a translational system will be impacted by complications after spinal cord injury such as muscle weakness and spasticity.

During functional movements, the wrist assists with grasping, for example grasp strength increases at a slight extension (~30 degrees from center [54]) and flexing the wrist assists with grasp release. Wrist movements are also necessary for orienting to objects or manipulating objects once grasped, for example pinch grip is most often preceded or followed by wrist flexion and extension [55]. During FES testing, the evoked 2-DOF (finger and wrist flexion) ROM and evoked postures are significantly impacted by coupling in the 2-DOFs, primarily the large wrist movements caused by stimulating extrinsic finger muscles (Supplemental Fig. 8). Despite the lack of independence, a simple control algorithm was able to continuously control stimulation commands on both DOF to successfully acquire 2-DOF target postures in the closed-loop task. Target postures across the finger and wrist ROMs were able to be achieved, with the most difficulty coming from reaching combined finger flexion with wrist extension due to the biomechanical coupling of the joints. As grasping movements with wrist extension are particularly important during functional movements [7, 54], more care is needed when designing stimulation to flex the fingers when the wrist is extended. For example, utilizing a stimulation sequence with minimal finger flexor stimulation, increased wrist extensor stimulation, or even wrist stiffness through co-contracting flexors and extensors [56]. Preliminary results show wrist extension can stabilize the wrist during finger flexion with wrist pattern stimulation keeping wrist posture minimally altered due to finger pattern stimulation (Fig. 5), however the extent of stabilization is not thoroughly tested here.

Future work can aim to improve FES accuracy by integrating more effective FES methods with BMI control. Typical closed-loop FES control methods use both feedforward and feedback control to better match a desired trajectory. In brain-controlled FES, the desired trajectory is dictated by the decoded brain signals, updated every 32 ms in this work. This results in a flexible control signal that enables the user to be a controller in the system (with visual feedback). However, purely brain-based control can also result in inefficient or unrealistic trajectories, for example full finger extension is inefficient with a fully extended wrist due to the shortened finger extensor muscles and a similar posture can even be obtained with no wrist extensor stimulation due to the torque at the wrist generated by finger extensors. More recent closed-loop FES work utilizes biomechanically constrained and energy efficient trajectories [57] to more reliably obtain intended movements. More investigation would be needed to effectively combine these efficient FES obtainable trajectories with BMI decoded intended trajectories.

With regards to stimulation protocols and recruitment curves, we found that there was high variance in how well evoked movements matched the intended movement, as given by the stimulation command (Fig. 6B). These patterns relating stimulation to movement were designed by hand using single electrode characterizations and trial-and-error methods with little data to estimate movements from stimulation on combinations of electrodes. Better modeling or sampling of stimulation induced movements could assist in stimulation design. Typical closed-loop FES approaches will include a transform from kinematics to muscle activations, along with the inverse, such that error can be calculated with kinematics and control updates can be made to muscle activations. In this work, the muscle activations are abstracted out, and joint angles are used for both error and control updates which simplifies the control to match the BMI output. This results in increased errors when there are non-stationarities in the relationship between stimulation and muscle activations or between muscle activations and resulting movements. Using algorithms such as Bayesian optimization could help efficiently tune stimulation parameters to obtain muscle activations [58, 59]. Then matching desired movements to muscle activations, either by training a model to match empirical EMG activity [60], or biomechanical model muscle activations, or utilizing actual biomechanical models, could more accurately inform the stimulation necessary to achieve target movements.

Furthermore, the closed-loop controller used for FES in this work was very simplistic. In addition to better designed stimulation protocols, more sophisticated feedforward and feedback controllers could be used to update stimulation and better obtain the intended movements [61–66]. Feedback controllers have been used extensively in FES systems to produce more accurate and smooth trajectories. For example, with arm movements, PI, PD, and PID controllers have been used to adjust FES to match hand trajectories or arm joint angles [65, 67–69]. A tuned feedback controller would likely produce smoother trajectories and less oscillation near targets in the closed-loop FES control in this work. Feedforward control, particularly model predictive control, is also promising for adding models of the system dynamics and muscle activations to produce more realistic and obtainable movements [57, 70]. Model predictive control could likely reduce the probability of reaching stuck postures, such as the finger stimulation holding the wrist joint flexed (i.e. preventing wrist extension) in this work.

With respect to BMI, the successful BMI control with a linear model indicate that the BMI algorithm could identify separate control signals for both the wrist and fingers using electrodes targeted to hand array of motor cortex. However, the FES evoked movements on the 2-DOF are not completely independent. More work is needed to understand how well BMI systems could use increasingly successful neural network methods [71], or feature engineering [22, 72], to better predict the required stimulation, or if the systems would be more effective when relying on the user to re-aim their BMI control [22, 73, 74].

### Limitations

Throughout these experiments muscle fatigue has been a limiting factor. In intramuscular FES, fatigue is caused by a few factors. Large motor units that fatigue quickly are recruited before or with smaller fatigue resistant fibers, a phenomenon known as reverse recruitment. Fibers are also recruited at relatively high rates. In normal recruitment, a fused contraction is formed by many overlapping twitches offset in time. In FES, all nearby fibers are activated at once, so a fused contraction must be created by stimulating at a higher rate. Here, significant fatigue was observed in some muscles after as few as three minutes of stimulation (Fig. 6D-F). One additional limitation of this study was that different levels of stiffness were not explored. Decreasing stiffness can reduce fatigue by requiring less muscle to be activated to achieve a posture. Experimenters did, however, aim for minimal stiffness while designing the stimulation patterns.

More work is needed to integrate stimulation paradigms that can alleviate fatigue, such as using more electrodes to activate more of the muscle [75], preferentially stimulating fatigue resistant muscle, interleaving stimulation on multiple electrodes [76], or targeting muscle motor points more specifically into systems designed to restore selective and functional movements [77].

## Supplementary Information

Below is the link to the electronic supplementary material.

Supplementary Material 1. List of implanted electrodes and expected function. Table showing both Monkey R and Monkey N, the various electrodes received and the expected functions.

Supplementary Material 2. Performance metrics during all BMI control experiments. Table showing all 3 sessions.

Supplementary Material 3. Additional Movie 1 Closed-loop hand control and BMI control.

Supplementary Material 4. Additional Movie 2 Open-loop finger movements in different wrist postures.

Supplementary Material 5. Additional Movie 3 Closed-loop FES control of simultaneous finger and wrist movements.

Supplementary Material 6. Supplemental Figure 1 Example stimulation patterns. (A) Stimulation pattern used to control the finger DOF in one session with Monkey R. The stimulation commands from 0-255 were mapped directly to the desired flexion (0-100%). (B) Example stimulation pattern used to control the wrist DOF in the same session as (a) with Monkey R. Supplemental Figure 2 Example stimulation patterns in multiple sessions. (A) Stimulation pattern used to control the wrist DOF in example sessions with Monkey R. Each line style (dotted, dashed, and solid) indicates a separate session for a total of three sessions. Stimulation intensity is presented as pulse charge (i.e., pulse width * pulse amplitude). (B) Example stimulation pattern used to control the finger DOF in the same sessions as (A) with Monkey R. Supplemental Figure 3 Additional finger and wrist angle measurement methods. (A) Illustration of the manipulandum used to measure wrist and finger flexion during BMI experiments. (B) Picture of bend sensors taped onto the monkey’s hand during FES experiments. Bend sensors were used during closed loop FES experiments. Supplemental Figure 4 Example target locations used during the BMI task. Target Targets were presented in a center-out fashion requiring flexing or extending the fingers or wrist from center rest (0.5 flexion for both finger and rest. This resulted in a total of 8 movement combinations and 3 different magnitudes of movement (20%, 30%, and 40% of calibrated range). These example targets are 18.8% of the wrist movement range and 16.9% of the finger movement range. Supplemental Figure 5 Illustration of the ReFIT data augmentation process. This illustration shows how predicted velocities used during closed-loop BMI control with the Kalman filter would be augmented before training the ReFIT Kalman filter. In this example which shows velocities in one time bin, the predicted wrist velocity is towards flexion however the target is towards extension. Assuming the intention is to move towards the target, the predicted wrist velocity will be adjusted by rotating it towards extension. On the other hand, since vectors are not rescaled, the finger vector, which is correctly pointing towards flexion but with an incorrect magnitude, does not get adjusted. Supplemental Figure 6 Example measurements from testing stimulation throughout the wrist and finger pattern. (A) Measured wrist (left, yellow) and index MCP (right, blue) angles from testing the wrist stimulation pattern on this day with Monkey R. Squares indicate the resting position before the stimulation started. (B) Same as (A) but for the finger stimulation pattern. Supplemental Figure 7 Change in range of motion centroid between early and late trials. Change in centroid between the boundary of evoked postures (as in Figure 6D) during the first 3 minutes and last 3 minutes using 2 days for monkey R and four days for monkey N. Supplemental Figure 8 Postures achieved during the 2-DOF command sampling range of motion experiments. Points are colored by the average measured finger angle (left) or wrist angle (right) reached in the last 500ms of stimulation at the commands given by the x and y axis before it was changed. Results are for one example session with Monkey R. Supplemental Figure 9 Closed-loop FES trial MSE by target location. MSE between obtained wrist and finger extension (averaged in last 100 ms of trial) and the target location for that trial. Target locations are grouped into bins with 10% of the target range for each DOF matching the presentation in Figure 7B and 7C. Results are presented for all trials (left) or for only successful trials (right) and are split by monkey (top – Monkey R, bottom – Monkey N). Significance stars indicate significant change in MSE for trials in that target location with respect to all trials (two-sided two-sample t-test with false discovery rate correction p<0.05). Stars within a target square indicate significance in trials to that wrist and finger target combination. Stars outside of a row or column indicate significance in trials to the wrist (columns) or finger (rows) target.

## Data Availability

All data supporting the findings of this study are available within the paper and its Supplementary Information.
