# Supplementary material for: Functional electrical stimulation and brain-machine interfaces for simultaneous control of wrist and finger flexion
Source: J Neuroeng Rehabil. 2026 Aug 5;23:312. doi: 10.1186/s12984-026-02091-7 (PMC13725986; doi:10.1186/s12984-026-02091-7)
Supplement: Supplementary file 1 [file 12984_2026_2091_MOESM1_ESM.docx]

**Additional Table 1**. List of implanted electrodes and expected function

| **Monkey R Electrodes** | **Expected Function** |
| --- | --- |
| Flexor Digitorum Superficialis (FDS) | All finger flexion |
| Flexor Digitorum Profundus, Ulnar Site (FDPr) | Ring finger flexion |
| Flexor Digitorum Profundus Radial Site (FDPi) | Index finger flexion |
| Flexor Digitorum Profundus Ulnar Site (FDPrs) | Ring and small finger flexion |
| Flexor Carpi Radialis Proximal Site (FCRp) | Wrist flexion |
| Flexor Carpi Radialis Distal Site (FCRd) | Wrist flexion |
| Flexor Carpi Ulnaris Proximal Site (FCUp) | Wrist flexion and adduction |
| Flexor Carpi Ulnaris Distal Site (FCUd) | Wrist flexion and adduction |
| Extensor Digitorum Communis Ulnar Site (EDCrs) | Ring and small finger extension |
| Extensor Digitorum Communis Radial Site (EDCi) | Index finger extension |
| Extensor Digitorum Communis Middle Site (EDCall) | Middle, ring, and small finger extension |
| Extensor Digitorum Communis Proximal Site (EDCim) | Index and middle finger extension |
| Extensor Indicis Proprius (EIP) | Index finger extension |
| Extensor Carpi Radialis Brevis Proximal Site (ECRBp) | Wrist extension |
| Extensor Carpi Radialis Brevis Distal Site (ECRBd) | Wrist extension |
| Extensor Carpi Ulnaris (ECU) | Wrist ulnar deviation |
| **Monkey N Electrodes** | **Expected Function** |
| Extensor Indicis Proprius (EIP) | Index finger extension |
| Flexor Digitorum Profundus, targeting MRS (FDP) | Middle, ring, and small finger flexion |
| Extensor Digitorum Communis (EDC) | All finger extension |
| Extensor Carpi Radialis Brevis (ECRB) | Wrist extension |
| Flexor Carpi Ulnaris (FCU) | Wrist flexion and adduction |
| Flexor Carpi Radialis (FCR) | Wrist flexion |
| Flexor Digitorum Profundus radial proximal site (FDPip) | Index finger flexion |
| Flexor Digitorum Profundus radial distal site (FDPid) | Index finger flexion |
