# Supplementary material for: Functional electrical stimulation and brain-machine interfaces for simultaneous control of wrist and finger flexion
Source: J Neuroeng Rehabil. 2026 Aug 5;23:312. doi: 10.1186/s12984-026-02091-7 (PMC13725986; doi:10.1186/s12984-026-02091-7)
Supplement: Supplementary file 2 [file 12984_2026_2091_MOESM2_ESM.docx]

**Additional Table 2.** Performance metrics during all BMI control experiments

| **Sessions** | **No. Successful Trials** | **Success Rate (%)** | **Acquisition Time (ms)** | **Time to Target (ms)** | **Orbit Rate (%)** | **Orbit Time (ms)** |
| --- | --- | --- | --- | --- | --- | --- |
| Session 1 |  |  |  |  |  |  |
| Hand Control | 583 | 96.2 | 804 | 570 | 48.4 | 473 |
| KF Control | 353 | 97.8 | 1131 | 683 | 56.7 | 864 |
| RKF Control  (Pre-block) | 257 | 97.7 | 1195 | 683 | 59.9 | 784 |
| RKF Control  (Post-block) | 135 | 84.9 | 1387 | 1067 | 43.0 | 624 |
| ReRKF Control | 159 | 94.6 | 907 | 779 | 32.1 | 736 |
| Session 2 |  |  |  |  |  |  |
| Hand Control | 375 | 94.0 | 1073 | 695 | 41.3 | 1350 |
| KF Control | 282 | 95.3 | 1131 | 811 | 47.5 | 768 |
| RKF Control  (Pre-block) | 145 | 98.0 | 971 | 843 | 37.2 | 912 |
| RKF Control  (Post-block) | 186 | 93.0 | 1611 | 939 | 55.4 | 1152 |
| ReRKF Control | 125 | 96.9 | 1003 | 715 | 52.0 | 512 |
| Session 3 |  |  |  |  |  |  |
| Hand Control | 431 | 99.1 | 738 | 581 | 27.8 | 679 |
| KF Control | 228 | 94.6 | 1522 | 859 | 60.1 | 1184 |
| RKF Control  (Pre-block) | 143 | 86.1 | 1227 | 779 | 51.7 | 768 |
| RKF Control  (Post-block) | 166 | 83.0 | 1650 | 1074 | 51.8 | 1040 |
| ReRKF Control | 156 | 94.0 | 1563 | 843 | 58.3 | 1376 |
