# Supplementary material for: Functional electrical stimulation and brain-machine interfaces for simultaneous control of wrist and finger flexion
Source: J Neuroeng Rehabil. 2026 Aug 5;23:312. doi: 10.1186/s12984-026-02091-7 (PMC13725986; doi:10.1186/s12984-026-02091-7)
Supplement: Supplementary file 3 [file 12984_2026_2091_MOESM6_ESM.docx]

**Supplemental Figure 1 – Example stimulation patterns.**

(**a**) Stimulation pattern used to control the finger DOF in one session with Monkey R. The stimulation commands from 0-255 were mapped directly to the desired flexion (0-100%). (**b**) Example stimulation pattern used to control the wrist DOF in the same session as (a) with Monkey R.

**Supplemental Figure 2 – Example stimulation patterns in multiple sessions.**

(**a**) Stimulation pattern used to control the wrist DOF in example sessions with Monkey R. Each line style (dotted, dashed, and solid) indicates a separate session for a total of three sessions. Stimulation intensity is presented as pulse charge (i.e. pulse width*pulse amplitude). (**b**) Example stimulation pattern used to control the finger DOF in the same sessions as (a) with Monkey R.

**Supplemental Figure 3 - Additional finger and wrist angle measurement methods.**

(**a**) Illustration of the manipulandum used to measure wrist and finger flexion during BMI experiments. (**b**) Picture of bend sensors taped onto the monkey’s hand during FES experiments. Bend sensors were used during closed loop FES experiments.

**Supplemental Figure 4 – Example target locations used during the BMI task.** Target Targets were presented in a center-out fashion requiring flexing or extending the fingers or wrist from center rest (0.5 flexion for both finger and rest. This resulted in a total of eight movement combinations and to three different magnitudes of movement (20%, 30%, and 40% of calibrated range). These example targets are 18.8% of the wrist movement range and 16.9% of the finger movement range.

**Supplemental Figure 5 – Illustration of the ReFIT data augmentation process.** This illustration shows how predicted velocities used during closed-loop BMI control with the Kalman filter would be augmented before training the ReFIT Kalman filter. In this example which shows velocities in one time bin, the predicted wrist velocity is towards flexion however the target is towards extension. Assuming the intention is to move towards the target, the predicted wrist velocity will be adjusted by rotating it towards extension. On the other hand, since vectors are not rescaled, the finger vector, which is correctly pointing towards flexion but with an incorrect magnitude, does not get adjusted.

**Supplemental Figure 6 – Example measurements from testing stimulation throughout the wrist and finger pattern. (a)** Measured wrist (left, yellow) and index MCP (right, blue) angles from testing the wrist stimulation pattern on this day with Monkey R. Squares indicate the resting position before the stimulation started. **(b)** Same as (a) but for the finger stimulation pattern.

**Supplemental Figure 7 – Change in range of motion centroid between early and late trials.**

Change in centroid between the boundary of evoked postures (as in figure 4(d)) during the first 3 minutes and last 3 minutes using two days for monkey R and four days for monkey N.

**Supplemental Figure 8 – Postures achieved during the 2-DOF command sampling range of motion experiments.** Points are colored by the average measured finger angle (left) or wrist angle (right) reached in the last 500ms of stimulation at the commands given by the x and y axis before it was changed. Results are for one example session with Monkey R.

**Supplemental Figure 9 – Closed-loop FES trial MSE by target location.** MSE between obtained wrist and finger extension (averaged in last 100 ms of trial) and the target location for that trial. Target locations are grouped into bins with 10% of the target range for each DOF matching the presentation in Figure 7B and 7C. Results are presented for all trials (left) or for only successful trials (right) and are split by monkey (top – Monkey R, bottom – Monkey N). Significance stars indicate significant change in MSE for trials in that target location with respect to all trials (two-sided two-sample t-test with false discovery rate correction p<0.05). Stars within a target square indicate significance in trials to that wrist and finger target combination. Stars outside of a row or column indicate significance in trials to the wrist (columns) *or* finger (rows) target.

| Monkey R Electrodes | Expected Function |
| --- | --- |
| Flexor Digitorum Superficialis (FDS) | All finger flexion |
| Flexor Digitorum Profundus, Ulnar Site (FDPr) | Ring finger flexion |
| Flexor Digitorum Profundus Radial Site (FDPi) | Index finger flexion |
| Flexor Digitorum Profundus Ulnar Site (FDPrs) | Ring and small finger flexion |
| Flexor Carpi Radialis Proximal Site (FCRp) | Wrist flexion |
| Flexor Carpi Radialis Distal Site (FCRd) | Wrist flexion |
| Flexor Carpi Ulnaris Proximal Site (FCUp) | Wrist flexion and adduction |
| Flexor Carpi Ulnaris Distal Site (FCUd) | Wrist flexion and adduction |
| Extensor Digitorum Communis Ulnar Site (EDCrs) | Ring and small finger extension |
| Extensor Digitorum Communis Radial Site (EDCi) | Index finger extension |
| Extensor Digitorum Communis Middle Site (EDCall) | Middle, ring, and small finger extension |
| Extensor Digitorum Communis Proximal Site (EDCim) | Index and middle finger extension |
| Extensor Indicis Proprius (EIP) | Index finger extension |
| Extensor Carpi Radialis Brevis Proximal Site (ECRBp) | Wrist extension |
| Extensor Carpi Radialis Brevis Distal Site (ECRBd) | Wrist extension |
| Extensor Carpi Ulnaris (ECU) | Wrist ulnar deviation |
| Monkey N Electrodes | Expected Function |
| Extensor Indicis Proprius (EIP) | Index finger extension |
| Flexor Digitorum Profundus, targeting MRS (FDP) | Middle, ring, and small finger flexion |
| Extensor Digitorum Communis (EDC) | All finger extension |
| Extensor Carpi Radialis Brevis (ECRB) | Wrist extension |
| Flexor Carpi Ulnaris (FCU) | Wrist flexion and adduction |
| Flexor Carpi Radialis (FCR) | Wrist flexion |
| Flexor Digitorum Profundus radial proximal site (FDPip) | Index finger flexion |
| Flexor Digitorum Profundus radial distal site (FDPid) | Index finger flexion |

**Supplemental Table 1 – List of implanted electrodes and expected function.**

|  | Number of successful trials | Success Rate (%) | Acquisition Time (ms) | Time to Target (ms) | Orbit Rate (%) | Orbit Time (ms) |
| --- | --- | --- | --- | --- | --- | --- |
| Session 1 |  |  |  |  |  |  |
| Hand Control | 583 | 96.2 | 804 | 570 | 48.4 | 473 |
| KF Control | 353 | 97.8 | 1131 | 683 | 56.7 | 864 |
| RKF Control  (Pre-block) | 257 | 97.7 | 1195 | 683 | 59.9 | 784 |
| RKF Control  (Post-block) | 135 | 84.9 | 1387 | 1067 | 43.0 | 624 |
| ReRKF Control | 159 | 94.6 | 907 | 779 | 32.1 | 736 |
| Session 2 |  |  |  |  |  |  |
| Hand Control | 375 | 94.0 | 1073 | 695 | 41.3 | 1350 |
| KF Control | 282 | 95.3 | 1131 | 811 | 47.5 | 768 |
| RKF Control  (Pre-block) | 145 | 98.0 | 971 | 843 | 37.2 | 912 |
| RKF Control  (Post-block) | 186 | 93.0 | 1611 | 939 | 55.4 | 1152 |
| ReRKF Control | 125 | 96.9 | 1003 | 715 | 52.0 | 512 |
| Session 3 |  |  |  |  |  |  |
| Hand Control | 431 | 99.1 | 738 | 581 | 27.8 | 679 |
| KF Control | 228 | 94.6 | 1522 | 859 | 60.1 | 1184 |
| RKF Control  (Pre-block) | 143 | 86.1 | 1227 | 779 | 51.7 | 768 |
| RKF Control  (Post-block) | 166 | 83.0 | 1650 | 1074 | 51.8 | 1040 |
| ReRKF Control | 156 | 94.0 | 1563 | 843 | 58.3 | 1376 |

**Supplemental Table 2 – Performance metrics during all BMI control experiments**

**Supplemental Movie 1 – Closed-loop hand control and BMI control.**

**Supplemental Movie 2 – Open loop finger movements in different wrist postures**

**Supplemental Movie 3 – Closed-loop FES control of simultaneous finger and wrist movements.**
